# National Center for Health Statistics Data Linkage Program: Generating Synthetic Data to Support Tiered Access.

**DOI:** 10.23889/ijpds.v9i5.2573

**Published:** 2024-09-18

**Authors:** Cordel Golden

**Affiliations:** 1 National Center for Health Statistics, Centers for Disease Control and Prevention

The National Center for Health Statistics (NCHS) has a long-established data linkage program that links NCHS health survey data with vital and other administrative records to expand the analytic potential of both the survey and administrative data. Integrating survey and administrative data provides a powerful mechanism for creating policy-relevant data resources to support the examination of factors that can influence disability, chronic disease, health care utilization, morbidity, and mortality. However, data privacy concerns impact the accessibility of NCHS linked data and thus its utilization. Nearly all NCHS linked data files are available only through the NCHS and Federal Statistical Research Data Centers. To minimize this barrier to access, the NCHS Data Linkage Program is engaged in a pilot project to create NCHS' first public-use fully synthetic data files. This initiative supports Evidence Act requirements, including tiered access to federal data. The talk will include an overview of the steps taken to generate the synthetic linked data and provide a verification process to support its analytic utility. The talk will also include a summary of NCHS’ exploration of other privacy enhancing technologies to increase partnership and data access.

